# DFT study of adsorbing SO_2_, NO_2_, and NH_3_ gases based on pristine and carbon-doped Al_24_N_24_ nanocages

**DOI:** 10.1007/s00894-023-05547-y

**Published:** 2023-04-14

**Authors:** R. A. Taha, A. S. Shalabi, M. M. Assem, K. A. Soliman

**Affiliations:** Department of Chemistry, Faculty of Science, Benha University, P.O. Box 13518, Benha, Egypt

**Keywords:** Al_24_N_24_ and Al_24_N_23_C nanocage, Toxic gases (SO_2_, NO_2_, And NH_3_), Adsorption, DFT

## Abstract

The adsorption of SO_2_, NO_2_, and NH_3_ toxic gases on Al_24_N_24_ and Al_24_N_23_C nanocages was investigated by using density functional theory (DFT) calculations. The adsorption energies, frontier orbitals, charge transfer using natural bonding orbital (NBO) analysis, dipole moment, the partial density of states (PDOS), thermodynamic relationships, non-covalent interaction (NCI), and quantum theory of atoms in molecules (QTAIM) were considered. The results reveal that carbon-doped Al_24_N_24_ nanocage increases the adsorption energies for SO_2_ and NO_2_ gases while decreasing the adsorption energy of NH_3_ gas. The Δ*G* for all configurations were negative except the configurations A1 and G2 confirming the weak adsorption of these two complexes. In conclusion, Al_24_N_24_ and Al_24_N_23_C nanocages are in general promising adsorbents for the removal of SO_2_, NO_2_, and NH_3_ toxic gases. The Al_24_N_24_ and Al_24_N_23_C nanocages are ideal electronic materials.

## Introduction

Sulfur dioxide (SO_2_), ammonia (NH_3_), and nitrogen dioxide (NO_2_) are toxic gases that pollute the environment [1, 2]. SO_2_ gas is produced by burning petroleum sources and fossil fuels. We can use it in many applications, but it has many problems for human health and causes many diseases such as lung disease, and a high dose of it causes coma or death [3–5]. NH_3_ is a colorless gas produced naturally from burning fossil fuels and when used in industry such as plastic production and preparation of nitric acid, it causes many problems such as eye and nose irritation, and a high dose of it may lead to death too [6]. NO_2_ is a reddish-brown gas produced by engine combustion producing acid rings, and when it reacts with water, it causes problems to the environment such as damaging buildings and many health hazards [7]. Therefore, the search for new materials to use as sensors that can detect and absorb these gases is aimed. There are several nanostructured materials in zero dimensions (0D), 1D, 2D, and 3D such as nanotubes, nanocages, and nanosheets that have been studied as gas sensors to detect these gases [8–28]. Derdare et al. used C_20_ and MC_19_ (M = Ru, Ir, and Au) clusters to adsorb and detect NO_2_, N_2_O, and NH_3_ gases by using DFT calculations, and their work showed that adsorption of N_2_O and NH_3_ on C_20_ is physical adsorption while NO_2_ is chemical adsorption, but the change of Eg is very low after adsorption indicating that the sensitivity of C_20_ to these gases is very low. While the doped form of MC_19_( RuC_19_ and AuC_19_) sensitivity and stability was increased, the IrC_19_ stability decreased. They also found that it is active and can be used as a catalyst in the decomposition of N_2_O gas [29]. Zahedi and Seif [30] show by using DFT calculations that C_48_B_6_N_6_ heterofullerene is a good material for use as a gas sensor to detect and adsorb NO_2_ and NH_3_ gases. Rad e al. [31] used pristine graphene (PG) and *N*-doped graphene to adsorb and detect SO_2_ and SO_3_ gases. They used DFT and NBO calculations and showed that adsorption energy in the case of NDG was higher than in pristine graphene; consequently, NDG is suitable and more detectable for these gases than pristine graphene. Basharnavaz et al. [32] used the P-doped and transition metal (TM)/P-codoped graphitic carbon nitride (gCN) systems (TM = Co, Rh, and Ir elements) to adsorb and detect SO_2_ gas. This work by using DFT calculations showed that TM/P-codoped gCN systems have adsorption energy higher than that of the pristine gCN. Also, they found that the Ir/P-codoped gCN was a better system for adsorption and detection of the SO_2_ gas compared with other systems with a high adsorption energy of − 3.52 eV. Zhang et al. [27] used graphene for the adsorption of SO_2_ by using DFT calculations. They showed that there were four structures for graphene, namely, pristine graphene (PG), vacancy-defected graphene (VG), Ti-doped graphene (Ti-G), and Ti-doped graphene with vacancies (Ti-VG). Their results showed that Ti-doped graphene is the best structure for the adsorption of SO_2_ gas with the highest adsorption energy. Noei [33] explained the electronic properties of pristine Al_12_N_12_ and B_12_N_12_ nanoclusters for the adsorption of SO_2_ gas. The results show that the two structures are sensitive to adsorb SO_2_ gas. Rad and Ayub [34] study the adsorption of O_3_ and SO_2_ molecules on pristine B_12_N_12_ and Ni-decorated B_12_N_12_ nanocages. They showed that the Ni-decorated B_12_N_12_ enhances and increases the adsorption of these gases. Xi et al. [35] examined the adsorption of CO_2_ molecules on two stable BiC and Bi2C monolayers promising adsorbents to capture CO_2_ gas. Zhao et al. [36] proposed a single metal catalyst on a 2D BC3N2 substrate for the activation of CO_2_ and CH_4_ gasses into CH_3_COOH. Huo et al. [37] used Fe_36_Co_44_ nanostructure to catalyze the hydrolysis reaction of ammonia borane to produce H_2_. Zhang et al. [38] study the removal of COS chemicals by nano-hollow sphere hydrolytic catalyst. Zhao e al. [39] investigated the 2D-InSe with B-doped as bifunctional catalysts to separate CO_2_ and CH_4_ under the regulation of an external electric field.

In this work, we study the adsorption of three toxic gases on Al_24_N_24_ and Al_24_N_23_C nanocages by using DFT calculations to decrease air pollution. The adsorption energies, NBO, PDOS, NCI, and QTAIM were determined.

## Computational details

In this study, all the geometries of nanocages and gas molecules before and after adsorption on nanocages were fully optimized based on density functional theory (DFT) without any symmetry constraint. The B3LYP and 6-31 g(d) level of theory were used for NO_2_ and SO_2_ systems and 6-31 g(d,p) for NH3 systems. The calculations were carried out by Gaussian 09 [40]. The adsorption energy of a gas molecule on the nanocages was obtained from the following equation:

1 $${E}_{ads}={E}_{gas@nanocage}-{E}_{gas}-{E}_{nanocage}$$

where *E*_*gas@nanocage*,_ *E*_*gas*_, and *E*_*nanocage*_ are the total energy of the gas molecule on the nanocage (Al_24_N_24_ and Al_24_N_23_C), the total energy of the gas molecule, and the total energy of the nanocage (Al_24_N_24_ and Al_24_N_23_C), respectively.

Thermodynamic parameters at *T* = 298.15 K and *P* = 1 atm such as Gibbs-free energy change (Δ*G*), enthalpy change (Δ*H*), and entropy change (Δ*S*) of the adsorption were evaluated.

## Results and discussion

### Geometry analysis

**Fig. 1:**
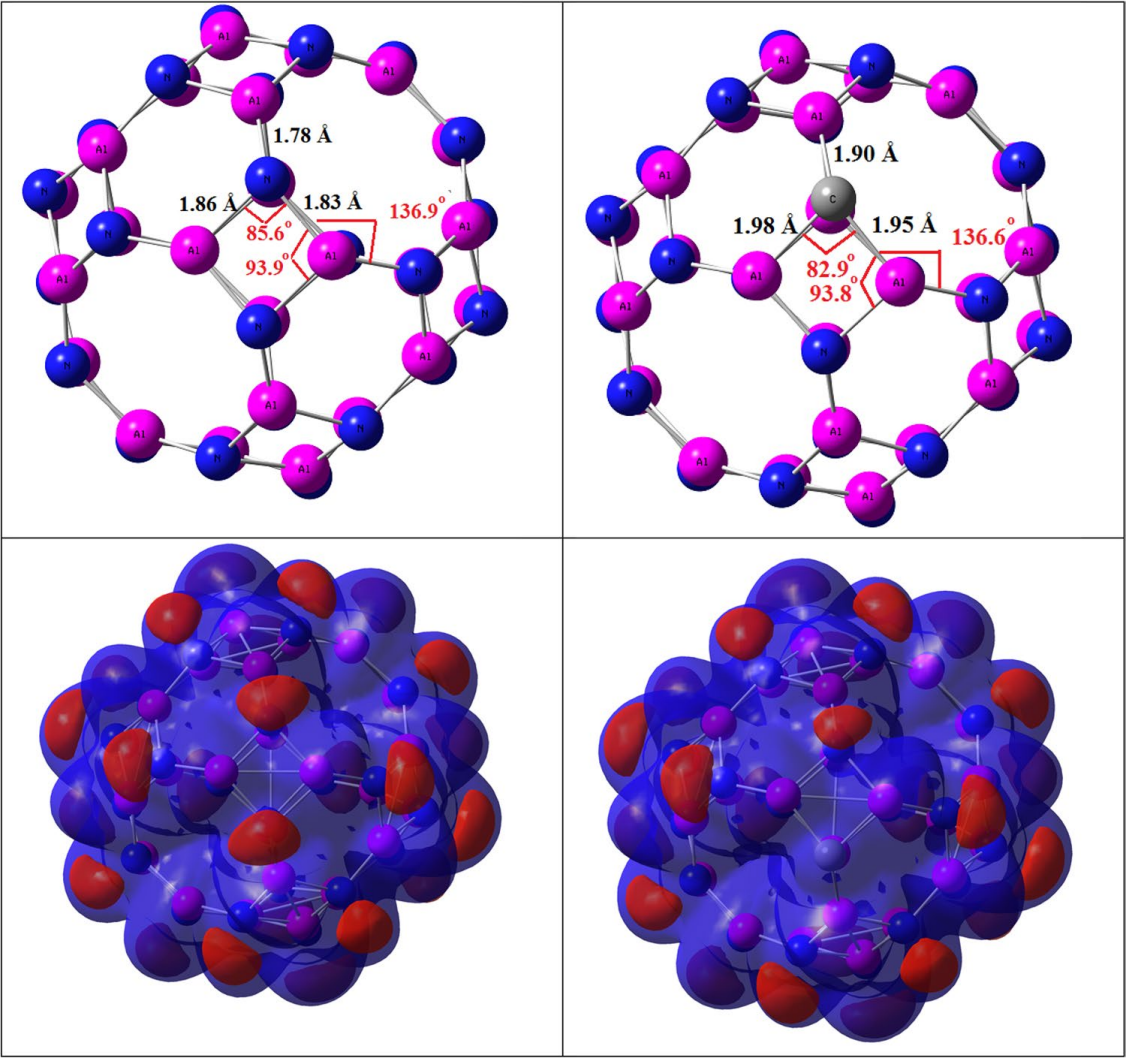
The optimized geometries and electrostatic potential map (ESP) of Al_24_N_24_ and Al_24_N_23_C

The adsorption capacity, which is one of the most important parameters that determine catalytic activity, indicates the selectivity of a particular substance by comparing it with other materials [41–43]. In this work, theoretical methods were used to study the adsorption of three industrial gases NO_2_, SO_2_, and NH_3_ based on the adsorbent material Al_24_N_24_ nanocage. As evident from Fig. 1, the nanocage of Al_24_N_24_ consists of 12 tetragons, 8 hexagons, and 6 octagons. As seen in Fig. 1, in pristine Al_24_N_24_, the bond distances between nitrogen and aluminum that are shared between 6- and 8-membered rings are 1.78 Å, 4- and 8-membered rings are 1.83 Å, and for 4- and 6-membered rings are 1.86 Å. By the replacement of one nitrogen with one carbon atom (Al_24_N_23_C nanocage), the bond distance between the doping atom and aluminum atom increases by 0.11 and 0.12 Å in comparison with pristine Al_24_N_24_.

Electrostatic potential map (ESP) is a useful tool for predicting the reactivity sites for a nucleophilic and electrophilic attack. As shown in Fig. 1, the red and blue colors represent the regions of negative (related to electrophilic reactivity) and positive (related to nucleophilic reactivity) electrostatic potential respectively. The negative regions are localized on the N atom, and the positive regions of ESP are localized on Al and C atoms.

### Interaction of NO_2_, SO_2_, and NH_3_ with Al_24_N_24_ and Al_24_N_23_C nanocages

**Fig. 2:**
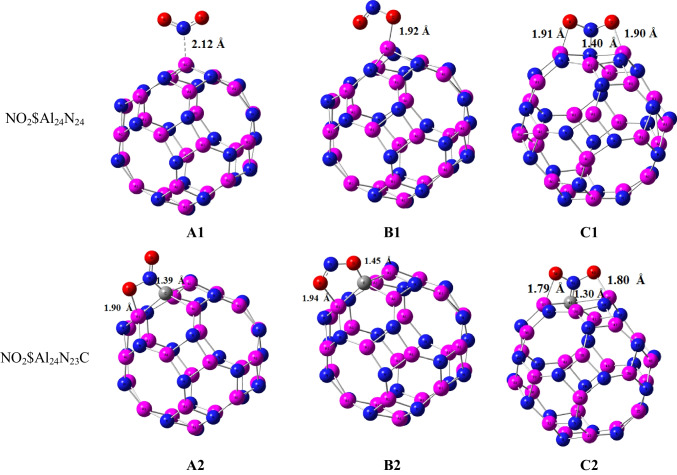
Adsorption configurations of NO_2_ on Al_24_N_24_ and Al_24_N_23_C

The optimized geometry configurations of NO_2_ on Al_24_N_24_ and Al_24_N_23_C nanocages are shown in Fig. 2. The three different configurations of NO_2_ on Al_24_N_24_ are represented as A1 to C1 and A2 to C2 for Al_24_N_23_C nanocages. Configuration A1 displays a nitrogen atom in a NO_2_ molecule interacting with an aluminum atom of pristine AL_24_N_24_ nanocage presented in Table 1, by distance 2.12 Å and adsorption energy − 0.19 eV. In complex A2, the NO_2_ molecule interacting with the doped carbon Al_24_N_24_ nanocage is different from that in configuration A1; the NO_2_ molecule interacts through nitrogen with carbon-doped Al_24_N_24_ and oxygen with the aluminum atom of Al_24_N_23_C. The additional interaction C of figuration A2 is responsible for strong binding energy by forming a C–N bond with a distance of 1.39 Å and the adsorption energy − 3.65 eV. A comparison between complex A1 and B1 indicates that the oxygen atom in the NO_2_ molecule strongly interacts with pristine Al_24_N_24_ nanocage as in complex B1, while in complex B2, by carbon-doped Al24N24, the interaction of nitrogen and oxygen atoms in NO_2_ is stronger than the two oxygen atoms of NO_2_ molecule. As seen in Table 1, the results reveal that adsorption energies are enhanced by carbon doping Al_24_N_24_. As shown in complex C1 and C2 in Fig. 2, the interaction of nitrogen in NO_2_ molecule is attracted by nitrogen of pristine Al_24_N_24_ and carbon of Al_24_N_23_C leading to the formation of N–N bond in pristine Al_24_N_24_ and C–N bond in Al_24_N_23_C nanocage. The bond lengths in both complex C1(N–N) and C2 (C-N) are 1.40 Å and 1.30 Å respectively.

**Table 1:** Structural parameters of NO_2_ adsorbed on AL_24_N_24_ and AL_24_N_23_C nanocage. The value between brackets is calculated by the cc-PVDZ basis set

	d (Å)	Eads (eV)	HOMO (eV)	LUMO (eV)	ΔE (eV)	µ (Debye)	Q_NBO_
Al_24_N_24_	–	–	− 6.48	− 2.39	4.09	0.0068	–
Al_24_N_23_C	–	–	− 6.35	− 2.34	4.01	0.6913	–
NO_2_$Al_24_N_24_ (A1)	2.12	− 0.19 (− 0.19)	− 6.40	− 2.48	3.92	4.2998	− 0.302
NO_2_$Al_24_N_23_C (A2)	1.39, 1.90	− 3.65 (− 3.51)	− 6.40	− 2.48	3.92	4.0570	− 0.467
NO_2_$Al_24_N_24_ (B1)	1.92	− 0.95 (− 0.91)	− 6.46	− 2.48	3.98	4.4967	− 0.61
NO_2_$Al_24_N_23_C (B2)	1.45, 1.94	− 3.14 (− 3.03)	− 6.31	− 2.75	3.56	2.7965	− 0.374
NO_2_$Al_24_N_24_ (C1)	1.90, 1.40, 1.91	− 1.41 (− 1.33)	− 6.42	− 2.43	3.99	3.9002	0.47
NO_2_$Al_24_N_23_C (C2)	1.80, 1.30, 1.79	− 3.83 (− 3.66)	− 6.31	− 2.47	3.84	3.7162	− 1.007

**Fig. 3:**
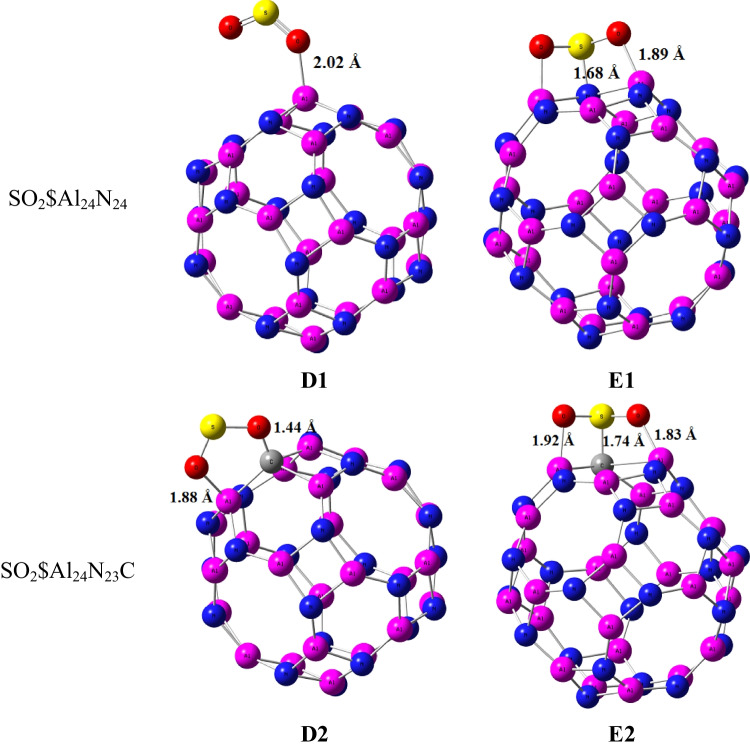
Adsorption configurations of SO_2_ on Al_24_N_24_ and Al_24_N_23_C

For SO_2_ gas adsorbed on the two nanocages Al_24_N_24_ and Al_24_N_23_C, the most stable orientations D1 and E1 for Al_24_N_24_, D2, and E2 for Al_24_N_23_C nanocages are depicted in Fig. 3. The adsorption energies for SO_2_ molecules in orientations D1 and D2 as shown in Table 2 are − 0.58 eV and − 2.10 eV, respectively. The interactions of the complex D1 and D2 are different like complex B1 and B2 of NO_2_ adsorbed on Al_24_N_24_ and Al_24_N_23_C. The presence of carbon doped in Al_24_N_24_ leads to improving the adsorption energy by about 3.62 times Al_24_N_24_ nanocage. The interaction of the sulfur atom in the SO_2_ molecule with the nitrogen of Al_24_N_24_ and carbon-doped Al_24_N_23_C is stronger as in orientations E1 and E2. The adsorption energies of the complexes E1 and E2 are − 2.58 eV and − 3.10 eV respectively.

**Table 2:** Structural parameters of SO_2_ adsorbed on AL_24_N_24_ and AL_24_N_23_C nanocage. The value between brackets is calculated by the cc-PVDZ basis set

	d (Å)	Eads (eV)	HOMO (eV)	LUMO (eV)	ΔE (eV)	µ (Debye)	Q_NBO_
SO_2_$Al_24_N_24_ (D1)	2.02	− 0.58 (− 0.73)	− 6.20	− 4.97	1.23	5.1966	0.06
SO_2_$Al_24_N_23_C (D2)	1.44, 1.88	− 2.10 (− 2.41)	− 5.73	− 2.33	3.40	3.0320	− 0.501
SO_2_$Al_24_N_24_ (E1)	1.68, 1.89	− 2.58 (− 2.79)	− 6.34	− 2.40	3.94	3.3922	− 0.3
SO_2_$Al_24_N_23_C (E2)	1.92, 1.74, 1.83	− 3.10 (− 3.31)	− 6.28	− 2.36	3.92	3.5360	− 0.494

**Fig. 4:**
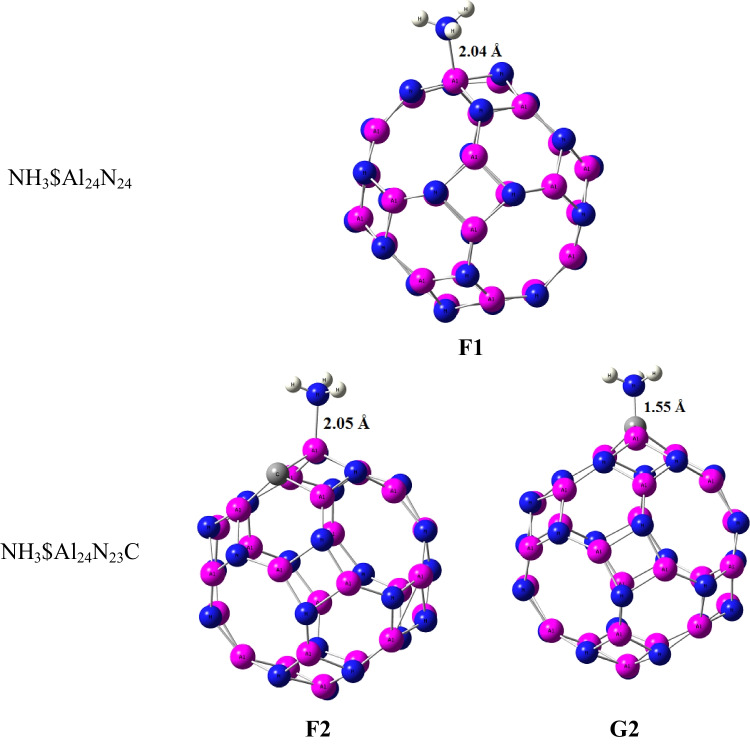
Adsorption configurations of NH_3_ on Al_24_N_24_ and Al_24_N_23_C

The response of carbon-doped Al_24_N_24_ nanocage is decreased towards the NH_3_ gas molecule. As shown in Fig. 4, there are three configurations for the adsorption of NH_3_ gas on Al_24_N_24_ and carbon-doped Al_24_N_23_C, the configuration F1, and the NH_3_ molecule prefers to lie on the aluminum atom of Al_24_N_24_ with adsorption energy − 1.41 eV as in Table 3. In configurations F2 and G2, NH_3_ binds with aluminum and carbon of Al_24_N_23_C, and the calculated adsorption energies for the complex F2 and G2 are − 1.36 eV and 0.41 eV showing that the interaction of NH_3_ with the carbon of Al_24_N_23_C is physical adsorption.

**Table 3:** Structural parameters of NH_3_ adsorbed on AL_24_N_24_ and AL_24_N_23_C nanocage. The value between brackets is calculated by the cc-PVDZ basis set

	d (Å)	Eads (eV)	HOMO (eV)	LUMO (eV)	Δ*E* (eV)	*µ* (Debye)	Q_NBO_
NH_3_$Al_24_N_24_ (F1)	2.04	− 1.41 (− 1.4)	− 6.11	− 2.13	3.97	6.7408	0.13
NH_3_$Al_24_N_23_C (F2)	2.05	− 1.36 (− 1.35)	− 5.90	− 2.08	3.82	7.4192	0.137
NH_3_$Al_24_N_23_C (G2)	1.55	0.41 (0.36)	− 6.11	− 2.24	3.87	6.7271	0.485

When the gas is adsorbed physically, we can reuse the substrate, while if a chemical bond is formed between the gas molecule and the substrate; it means that the desorption process is difficult. As presented in Tables 1 and 2, the doped Al_24_N_24_ by carbon atom for SO_2_ and NO_2_ molecules is chemically adsorbed, so this nanocage is not suitable for sensing SO_2_ and NO_2_ gas molecules. In the case of NH_3_ systems, the binding of the NH_3_ gas molecule with the aluminum atom as seen in Table 3 configuration F2 is lower in adsorption energy than configuration F1. As revealed from their results, pristine AL_24_N_24_ nanocage is sensitive to the three gas molecules while carbon-doped (Al_24_N_23_C) nanocage is sensitive only to NH_3_ gas molecule. We propose a diagnostic test for the effect of the basis set by using cc-PVDZ as a single-energy calculation, as seen in Tables 1, 2, and 3; the calculated adsorption energies show change ranges from 0 to 0.17 eV in the case of NO_2_, from 0.11 to 0.31 eV for SO_2_, and from 0.01 to 0.05 eV for NH_3_ as we compare with the basis set used for this study mentioned in computational details.

### NBO charges and dipole moment

**Fig. 5:**
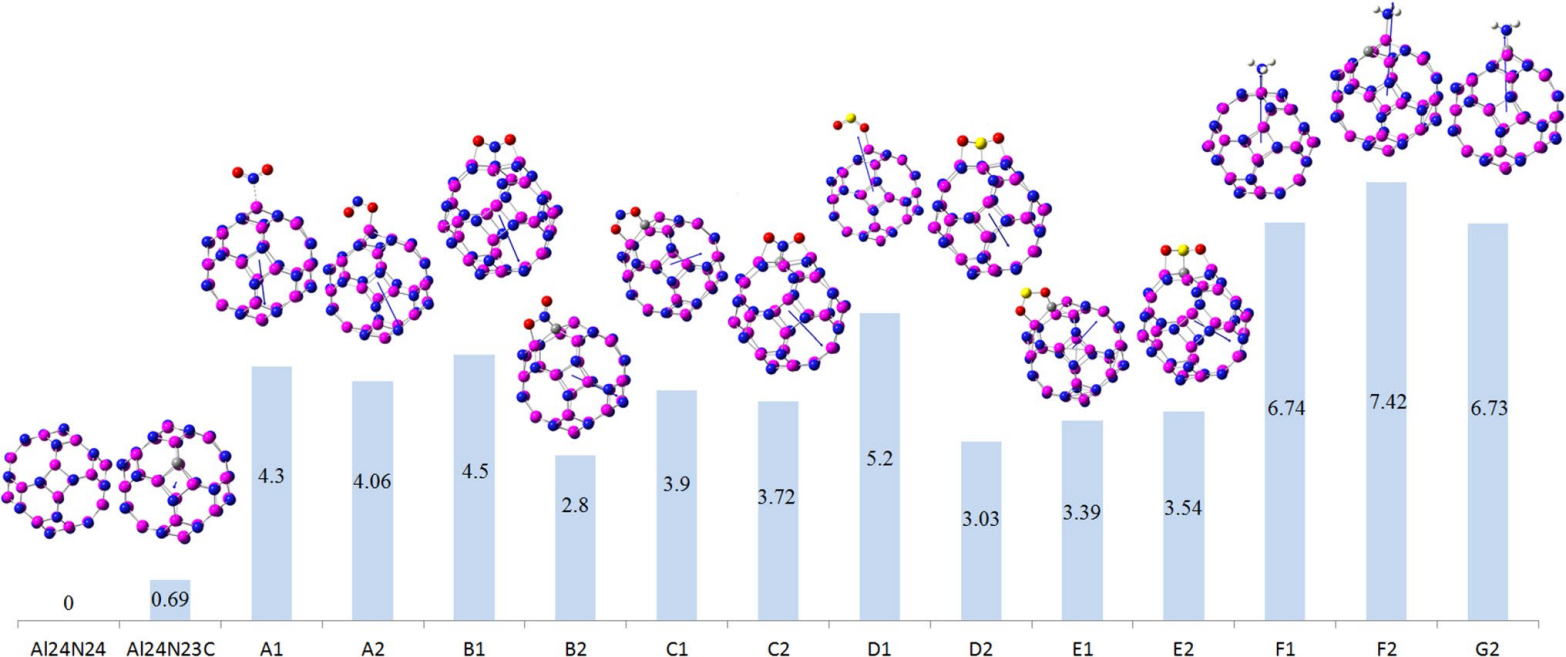
Dipole moment directions for the two nanocages together with the adsorbed gas molecules

The dipole moment and the charge transfer for the adsorbed gases on pristine Al_24_N_24_ and carbon-doped Al_24_N_23_C nanocages were investigated. As seen in Table 1, the dipole moment of the pristine Al_24_N_24_ nanocage is 0.0068 Debye. After doping with carbon, the dipole moment was changed to 0.6913 Debye. The adsorption of NO_2_, SO_2_, and NH_3_ gases on the two nanocages brings a change in the dipole moment. As seen in Fig. 5, the dipole moment vectors of the SO_2_ and NO_2_ adsorbed on Al_24_N_24_ and Al_24_N_23_C nanocages point away from these two groups. For configuration D1 where the SO_2_ group is adsorbed on Al_24_N_24_ nanocage, the dipole moment vector points forward to this group. For the NH_3_ group adsorbed on Al_24_N_24_ and Al_24_N_23_C nanocages, the directions of dipole moment vectors point forward to this group. The charge transferred was determined for all complexes. As seen in Tables 1, 2, and 3, the charge transfers from the nanocage to the SO_2_ and NO_2_ gas molecules due to the withdrawing nature of the SO_2_ and NO_2_ molecules. For configuration C1, this is due to N–N bond formation between NO_2_ gas and Al_24_N_24_ nanocage and for configuration D1 because SO_2_ binds to the Al_24_N_24_ through an oxygen atom of SO_2_ molecule. As a comparison between configuration A2 and C2 where NO_2_ adsorbs on carbon-doped nanocage (Al_24_N_23_C), the value of charge transferred for configuration C2 is larger than in configuration A2; this indicates stronger interaction between NO_2_ and nanocage in configuration C2 than that of configuration A2. Note that when the value of charge transfer increases, a strong interaction between gas molecules and substrates occurs. For NH_3_ systems, the charge is transferred from the gas molecule to the nanocage.

### Thermodynamic parameters and density of states

The analysis of interaction energies is expanded to include enthalpies (*H*), Gibbs-free energies (*G*), and entropies (*S*) of interaction. The thermodynamic parameters for the adsorbed gases SO_2_, NO_2_, and NH_3_ such as Gibbs-free energy change (Δ*G*), enthalpy change (Δ*H*), and entropy change (Δ*S*) were determined at *T* = 298.15 K and *P* = 1 atm.

As seen in Table 4, the negative values of Gibbs-free energies indicate the spontaneous adsorption of gases molecule on the two nanocages. The higher value of Δ*G* implies a strong interaction between the gas molecule and substrate. The enthalpy change values are higher than the Gibbs-free energy values which indicate that the enthalpy is stabilized. The Δ*G* for the configurations A1 and G2 have a positive value confirming weak adsorption for these complexes.

**Table 4:** Thermodynamic parameters of the gas molecules adsorbed on nanocages

	Δ*G* (Kcal/mole)	Δ*H* (Kcal/mole)	Δ*S* (Cal/mole-K)
NO2$Al24N24 (A1)	5.81	− 3.52	− 31.295
NO2$Al24N23C (A2)	− 68.02	− 81.77	− 46.098
NO2$Al24N24 (B1)	− 10.67	− 21.40	− 35.98
NO2$Al24N23C (B2)	− 56.63	− 70.66	− 47.04
NO2$Al24N24 (C1)	− 17.85	− 31.39	− 45.41
NO2$Al24N23C (C2)	− 71.87	− 86.33	− 48.485
SO2$Al24N24 (D1)	− 2.45	− 12.34	− 33.19
SO2$Al24N23C (D2)	− 34.82	− 47.42	− 42.252
SO2$Al24N24 (E1)	− 44.34	− 58.25	− 46.67
SO2$Al24N23C (E2)	− 56.67	− 70.24	− 45.51
NH3$Al24N24 (F1)	− 20.55	− 30.36	− 32.89
NH3$Al24N23C (F2)	− 19.63	− 29.17	− 31.99
NH3$Al24N23C (G2)	21.91	11.52	− 34.86

In Tables 1, 2, and 3, the quantum parameters and the electronic properties of the gaseous molecule were evaluated. The HOMO of most complexes is shifted to higher energy. The HOMOs of adsorbed gases were comparable. After the adsorption of this NO_2_, SO_2_, and NH_3_ on the Al_24_N_24_ and Al_24_N_23_C, the energy gap for all systems decreased.

### The PDOS analysis

**Fig. 6:**
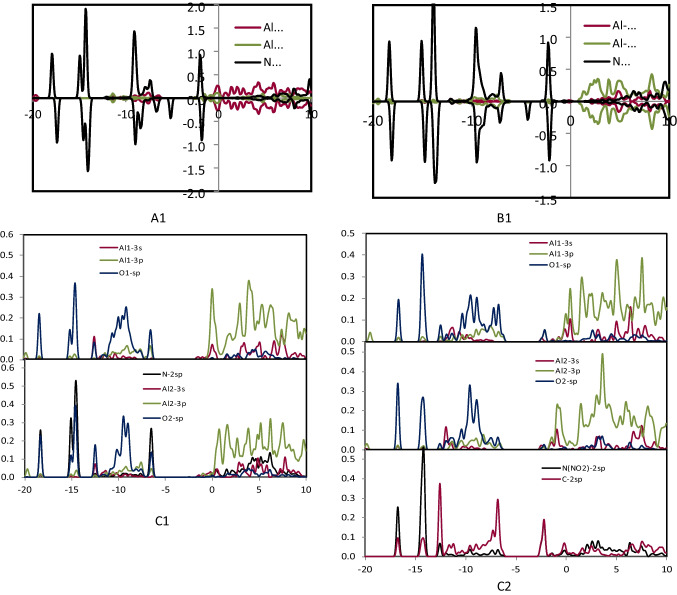
PDOS of configuration A1, B1, C1, and C2

**Fig. 7:**
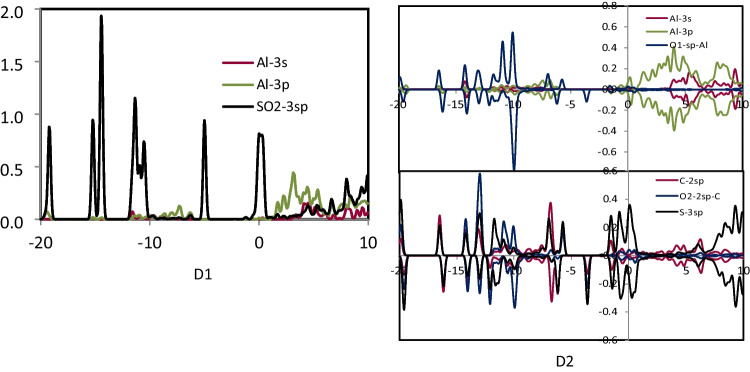
PDOS of configuration D1 and D2

**Fig. 8:**
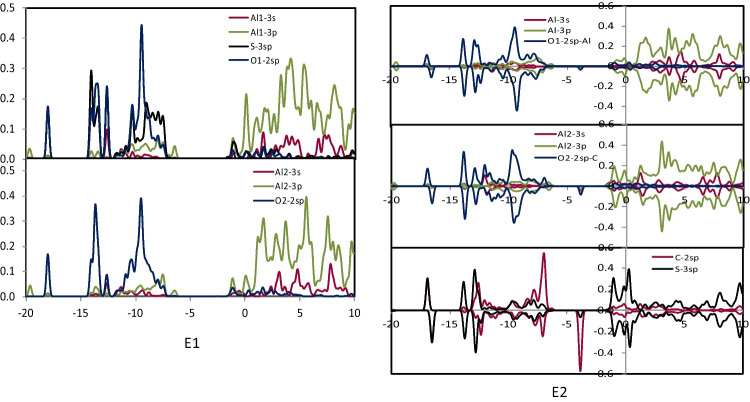
PDOS of configuration E1 and E2

**Fig. 9:**
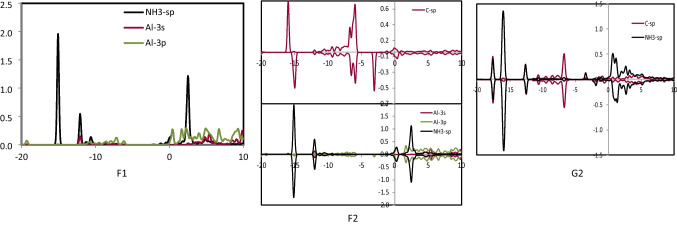
PDOS of configuration F1, F2, and G2

To characterize the interactions between SO_2_, NO_2_, and NH_3_ gases with the study nanocages (Al_24_N_24_ and Al_24_N_23_C), the projected density of states (PDOS) was examined. After adsorbing SO_2_, NO_2_, and NH_3_ gases on Al_24_N_24_ and Al_24_N_23_C, the PDOS of O-2sp, N-2sp, C-2sp, S-3sp, NH_3_-sp, Al-3 s, and Al-3p orbitals was displayed in Figs. 6, 7, 8, and 9. It is important to note that the charge transfers from Al to the anti-bonding states of NO_2_ and SO_2_ molecule that elongates the N–O and S–O bond to 1.341 Å, and 1.372 Å at C2 and E2, respectively. It was shown that the O-2sp, N-2sp, and S-3sp orbitals were found to be particularly localized close to the Fermi level; facilitating their interactions with the C-2sp, Al-3 s, and Al-3p states in Al_24_N_23_C nanocage indicates the NO_2_ and SO_2_ may have a higher binding energy (*E*_*b*_) if it is close to the C atom in the Al_24_N_23_C nanocage. Figure 6 shows that the Al-3 s and Al-3p orbitals are pushed to lower energies as a result of electron transfer from the Al atoms to the NO_2_ and SO_2_ molecules, which considerably aids in the adsorption of NO_2_ and SO_2_.

By the binding energies indicated in Tables 1 and 2, it is seen that the Al-3p orbitals of C2′s PDOS display increased intensity, indicating that C2 is more significant than both E2 and C1 configurations. Furthermore, from − 6.20 to − 12.70 eV, the N-2sp states of adsorbed NO_2_ contribute significantly to the C-2sp (HOMO), indicating that the C atom uses its valence orbitals to interact with the O-2sp, N-2sp, Al-3 s, and Al-3p states of NO_2_ as well as the Al_24_N_23_C nanocage. Due to the distribution of S-3sp states in a broad range from − 5.40 to − 14.30 eV (E2) below the Fermi level, the S-3sp orbitals of SO_2_ also show a stronger hybridization with the C-2sp, Al-3 s, and Al-3p orbitals of Al_24_N_23_C. Agreeing with their binding energies, the charge-transfer values between the S, N, and C atoms of NO_2_ and SO_2_ and the Al_24_N_23_C nanocage suggest that the charge-transfer is more important for the stability of these complexes than the electrostatic interactions. On the other hand, a weak connection between the substrate and the NH_3_ molecule results from the soft hybridization of the NH_3_ and Al_24_N_23_C′s C-2p orbitals.

### Non-covalent interaction (NCI) analysis

To identify non-covalent bonds, such as hydrogen bonds, steric clashes, Van der Waals (vdW), and reduced density gradients, the quantum mechanical electron density has been used (RDG) determined by the following equation:

2 $$RDG\left(r\right)=\frac{1}{2(3{\pi }^{2}{)}^{1/3}}\frac{\left|\nabla \rho (r)\right|}{\rho (r{)}^{4/3}}$$

where the $\rho$ value can represent the bond strength, whereas the value of sign(_2_) × $\rho$ is used to evaluate the nature of the interaction between the SO_2_, NO_2_, and NH_3_ gases and the investigated nanocages (Al_24_N_24_ and Al_24_N_23_C). _2_ is the second-largest eigenvalue of the electron density in the Hessian matrix, where sign(_2_) × $\rho$  < 0 denotes an attractive interaction and sign(_2_) × *ρ* > 0 denotes a repulsive interaction.

**Fig. 10:**
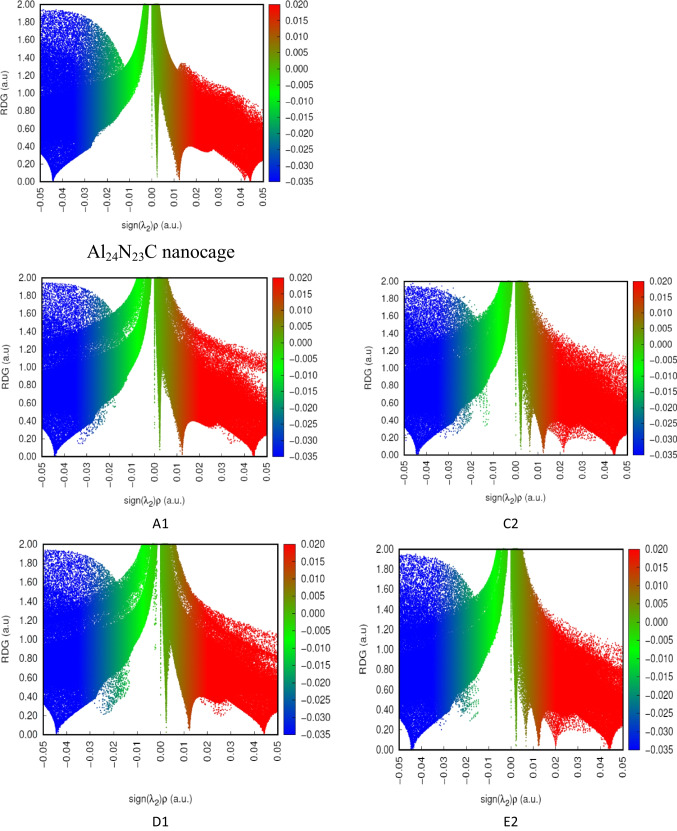
Non-covalent interaction of G2 and F1

**Fig. 11:**
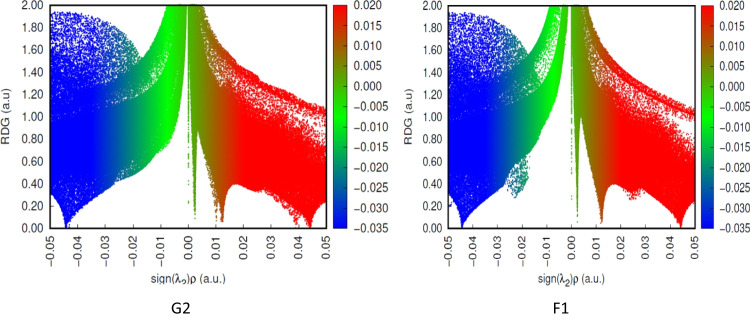
Non-covalent interaction of Al_24_N_23_C nanocage, A1, C2, D1, and E2

To study the non-covalent interactions between the NO_2_, SO_2_, and NH_3_ gases and the investigated nanocages, the scatter graphs between the reduced density gradient (RDG) and the electron density ($\rho$) have been shown in Figs. 10 and 11. As seen in Figs. 10 and 11, sign(λ_2_)$\rho$) increases for red areas suggesting high steric repulsions inside the nanocage, whereas sign(*λ*_2_)$\rho$ decreases for blue areas indicating strong contacts. The green areas between the complex’s elements approaching 0 are Van der Waals interactions, which are weak intermolecular forces. The considerable vdW interaction between the NH_3_ and the Al_24_N_23_C (G2) nanocages is visible, as can be seen in Fig. 11. This conclusion is supported by the geometric analysis, which considers weak interactions.

Additionally, blue and green blended spikes were seen at greater electron densities and sign(*λ*_2_)$\rho$< 0, which shows that the C2, E2, and F1 complexes have developed partial covalent connections. The blue and green patches between the gas molecule and the nanocages, as well as the intermolecular contact, were visible in the partial covalent bond as shown in Figs. 10 and 11. Higher interaction intensity was seen for C2 and E2 configurations due to the partial covalent contact between the SO_2_ and NO_2_ molecules with the Al_24_N_23_C nanocage in the RDG versus sign(_2_) × *ρ* graph.

### Quantum theory of atoms in molecules (QTAIM)

**Fig. 12:**
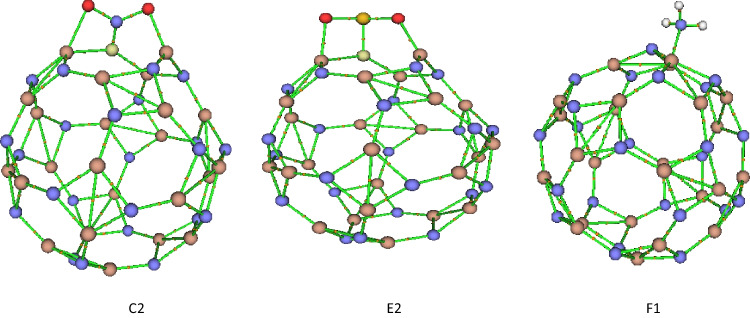
AIM molecular graphs for C2, E2, and F1

A well-known method for examining the topology of interactions is QTAIM (covalent or non-covalent). For the most stable configuration of C2, E2, and F1 complexes, measurements of several topological parameters, including electron density (*ρ*_*r*_), Laplacian (${\nabla }^{2}$ *ρ*_*r*_), and total electron energy density (*H*_*r*_) at the bond critical points (BCP), have been made in order to characterize the strength and type of bond (Table 5). Shared shell interactions (covalent bonds) exist when the *ρ*_*r*_ is larger than 0.20 a.u., is higher than 0.20 a.u, and the Laplacian is higher but negative. Al24N23C nanocage’s Al-to-carbon bond is demonstrated to be partially covalent in the presence of ${\nabla }^{2}$ *ρ*_*r*_ > 0 and *Hr* < 0 [44]. However, *ρ*_*r*_ < 0.1 a.u [45] indicates closed-shell interactions (ionic, hydrogen bonding, or van der Waals interactions). Figure 12 displays the BCP between the atoms of the complexes under investigation. As shown in Table 5, the results for the C2 complex indicate that the **N–C** bond should be regarded as a strong polar covalent bond because its *ρ*_*r*_ value is 0.326 a.u, which is somewhat higher than the **S–C** in E2. According to the binding energies, the Al–C (Al_24_N_23_C) bond in the C2 complex has a higher *ρ*_*r*_ value for BCP than the same bond in the E2 configuration. A weaker association between NH_3_ and nanocages is also indicated by the fact that F1′s electron density (0.050 a.u.) is lower than that of C2 and E2. The low values of *ρ*_*r*_ < 0.1 for Al–C, Al1–O, Al2–O, and Al–N bonds of SO_2_, NO_2_, and NH_3_ at Al_24_N_24_ and Al_24_N_23_C nanocages show that the charge dissipates in the distance between the two nuclei and that the interactions can be categorized as a closed-shell type, which is related to strong non-covalent interactions [46, 47].

**Table 5:** The topological parameters, including electron density (*ρ*_*r*_), Laplacian of electron density (${\nabla }^{2}$*ρ*_*r*_), the density of kinetic energy (*G*_*r*_), the density of potential energy (*V*_*r*_), and the density of total energy (*H*_*r*_) in (a.u.) at the bond critical point (BCPs) for the most stable configurations C2, E2, and F1

Structure	Bonds	*ρr*	${\nabla }^{2}$ *ρr*	Gr	Vr	Hr
C2	N–O	0.346	− 0.446	0.224	− 0.559	− 0.336
	N–O	0.378	− 0.540	0.254	− 0.644	− 0.389
	N–C	0.326	0.373	0.607	− 1.121	− 0.514
	Al1–O	0.072	0.502	0.122	− 0.118	0.004
	Al2–O	0.055	0.304	0.077	− 0.079	− 0.001
	Al–C	0.068	0.277	0.084	− 0.099	− 0.015
E2						
	Al–C	0.065	0.264	0.078	− 0.091	− 0.012
	Al1–O	0.061	0.389	0.095	− 0.093	0.002
	Al2–O	0.072	0.528	0.126	− 0.119	0.006
	S–O	0.227	0.112	0.293	− 0.558	− 0.265
	S–O	0.207	− 0.043	0.221	− 0.453	− 0.232
	S–C	0.216	− 0.398	0.070	− 0.240	− 0.170
F1						
	Al–N	0.050	0.272	0.067	− 0.066	0.001
	N–H	0.337	− 1.792	0.050	− 0.549	− 0.498
	N–H	0.337	− 1.800	0.051	− 0.549	− 0.499
	N–H	0.338	− 1.802	0.050	− 0.550	− 0.500

## Conclusion

In this paper, the aim was to investigate the use of Al_24_N_24_ and Al_24_N_23_C nanocages toward three harmful gases (NO_2_, SO_2_, and NH_3_). The adsorption properties were determined through adsorption energies, charge transfer, dipole moment, thermodynamic parameters, PDOS, NCI, and QTAIM, and the following results are obtained:

- Introducing carbon-doped increases the adsorption energies for NO_2_ and SO_2_ gases, while decreasing for NH_3_ gas
- The charge is transferred from the NH_3_ gas molecule to the nanocage, while for NO_2_ and SO_2_ systems, the charge transfers from the nanocage to the SO_2_ and NO_2_ gas molecules except configurations C1 and D1.
- The directions of dipole moment vectors for the NH_3_ system point forward to this group, while dipole moment vectors of the SO_2_ and NO_2_ adsorbed on Al_24_N_24_ and Al_24_N_23_C nanocages point away from these two groups except configuration D1.
- The Gibbs-free energy change (Δ*G*) for all configurations is negative except that A1 and G2 have a positive value confirming weak adsorption for these complexes.
- The energy gaps decreased after adsorping NO_2_, SO_2_, and NH_3_ on Al_24_N_24_ and Al_24_N_23_C nanocages.
- Higher interaction intensity was observed for C2 and E2 configurations due to the partial covalent contact between the SO_2_ and NO_2_ molecules with the Al_24_N_23_C nanocage.
- The results obtained from QTAIM for the C2 complex indicate that the **N–C** bond should be regarded as a strong polar covalent bond because its *ρ*_*r*_ value is 0.326 a.u and a weaker association between NH_3_ and Al_24_N_24_ nanocages is indicated by electron density (0.050 a.u.) that is lower than that of configuration C2 and E2.

These results confirm that Al_24_N_24_ and Al_24_N_23_C nanocages were used as promising materials for the removal of NO_2_, SO_2_, and NH_3_ toxic gases.

## Data Availability

The authors confirm that the data supporting the findings of this study are available within the article. Code used for calculated data and analysis is commercial, and the authors have a license to the software.
